# Teacher Overinvolvement and Student Depression among Junior High School Students in Taiwan

**DOI:** 10.1100/tsw.2006.152

**Published:** 2006-07-21

**Authors:** Fen Fen Huang, Cou Chen Wu, Chang Ya Hu, Sun Shen Yang

**Affiliations:** ^1^ Department of Business Administration, National Taiwan University of Science and Technology, Taiwan; ^2^ Department of Community Medicine, Center for Occupational Disease, Taipei City Hospital, Taiwan

**Keywords:** teacher emotional overinvolvement, student depression, Taiwan

## Abstract

This study examines depression in students at public high schools in Taiwan. The purpose of this study is to examine which student-level and teacher-level variables affect student depression due to teacher emotional overinvolvement and other factors. A survey instrument adapted and translated from existing surveys was distributed to 1,479 Taiwanese adolescents aged 13—15 years and 172 teachers from 10 public junior high schools in the city of Taipei. The hierarchical linear model (HLM) was used for a cross-level analysis of the data. The HLM shows that student-level measures account for most of the variance. Teacher emotional overinvolvement and core self-evaluations are the preponderant influences on student ratings. In terms of teacher-level variables, the effects of teacher involvement, teacher depression, and teacher educational background on student-level variables are strong and significant. The findings of this study recommend the development of a comprehensive counseling system for teachers and students.

## INTRODUCTION

In the Taiwanese educational system, junior high school homeroom teachers have more authority and responsibility than their American counterparts. They are responsible for providing both educational and personal guidance to their students. Junior high school students face the unceasing character and behavioral changes of adolescence. In this period of personality development, their homeroom teacher can play a significant role. Because homeroom teachers wield a great deal of authority in Taiwan, a culture where respect for authority is strongly ingrained, they may be deeply involved in the lives of their individual students. This leads many students to experience increased pressure and to perceive that their own autonomy has been circumscribed.

Previous studies of teacher behavior have analyzed indirect influencing behavior such as teacher reception, reward, adoption of opinions from students, question asking, and warm-heartedness, as well as direct influencing behavior such as teacher classroom instruction, punishment, and authority[1,2,3]. Students do not always perceive teacher behavior as positive, mainly due to teacher emotional overinvolvement[4]. Students may either perceive teacher involvement as positive (teacher support)[5] or negative (teacher emotional overinvolvement). The present study uses student perceptions to explore the influence of teachers on students.

Teacher emotional overinvolvement is manifested when the teacher in charge of a class feels overly responsible for student performance, and is thus too concerned and too emotional with students. When students cannot reach the required level of performance, the teacher blames himself/herself for not being involved enough to the point of investing his or her personal emotional well-being in student performance. As a result, the most common effect of student dissatisfaction with teacher overinvolvement is depression. Students become passive, and open conflicts between students and teachers are rare because the teacher-student relationship is not based on reciprocity. Student expressions of dissatisfaction are limited by the expectations of traditional society.

Depression among adolescents is a serious issue in Taiwan, according to statistics. Among the top ten causes of adolescent death, suicide is the third[6]. Depression is an important factor leading to adolescent suicide[7,8,9]. The particular education system that incorporates elements of Chinese culture gives too much authority to teachers. Most teachers still have the right to impose corporal punishment, hair length restriction, and other regulations. The present study is interested in this particular cultural context, and seeks to understand the relationship between teachers and students in junior high schools. In the past, many studies explored the influence of parental emotional overinvolvement on childhood depression[10,11]. The present study explores the influence of homeroom teacher emotional overinvolvement on student depression within the distinct Taiwanese educational system. Furthermore, the study examines the effects of homeroom teacher depression on student depression.

## Literature Review

There are few countries where junior high school teachers have as a great power over and are as deeply involved in student lives as in Taiwan. Despite this fact, research on the topic of junior high school teacher overinvolvement with students is limited. Because of the paucity of research on this topic, the present study makes use of the research on the theory of "miscarried helping" from the study of Coyne et al.[4], and applies this theory to student-perceived teacher emotional overinvolvement in Taiwan. Coyne's study consisted largely of clinical observations on small focus groups, but this study tests a large sample. Consequently, Coyne's concept of miscarried helping is quantified in order to carry out additional research. A similar concept used as a variable in schizophrenia research called "expressed emotion", one of the indicators used to measure emotional overinvolvement of health care providers, is also used in the present study. The emotional overinvolvement scale consists of 45 items relating to the three behaviors that reflect emotional overinvolvement — overprotectiveness, overconcern, and overidentification[12,13]. Included within these items are the behaviors of emotional overreaction, interdependency, extreme devotion, and self-sacrifice. In this study, the first two behaviors (overprotectiveness and overconcern) are analyzed, but the third (overidentification) is not directly related to the study so is not investigated.

In Taiwan, depression is widespread among junior high school students. According to a Taipei sanatorium investigation, approximately 10% of adolescents in the city of Taipei present symptoms of depression, 25% of whom will become chronic mental-health care patients[14]. In addition, according to a study on risk and protective factors of adolescent depressive mood, it indicated 84.2% of teenagers in Taiwan have experienced feeling depressed and these teenagers believe the causes are academic demands, peer relationships, and studying for exams. [15]. As the facts above prove, adolescent depression in Taiwan cannot be ignored. Many child and adolescent epidemiology studies indicate that depression before 13 years of age is not common, but increases substantially after 13 years of age, peaking between 15 and 18 years of age. Many people experience their first depression in adolescence and then experience recurrences throughout their lives[16,17,18].

Shek et al. published on adolescent suicide from 1980–2003 in Hong Kong; there are several trends and patterns[19]:

1. There was a gradual increase in adolescent suicide rates in the 1990s and in the early 2000s, the rates were higher than those in the 1980s.
2. Suicide rates for adolescents aged 10–24 years were lower than those of other adult age groups.
3. Although adolescent suicide rates in Hong Kong were lower than those reported in some English-speaking countries and Mainland China, the figures were higher than those reported in Taiwan.
4. Suicide rates among teenagers in early adolescence were lower than those among adolescents in late adolescence.
5. Although male adolescent suicide rates were in general higher than female adolescent suicide rates among the 10–24 age groups, gender differences in suicide rates appeared to be driven by age.
6. There was a gradual rising trend in adolescent proportional mortality rates for suicide since the 1990s.
7. Proportional mortality rates for suicide among teenagers in early adolescence were lower than those among adolescents in late adolescence.

As discussed above, depression is an emotional disturbance that includes unhappiness, downcast mood, pessimism, helplessness, and may lead to suicide. While a slight short-term depression may help individuals adapt themselves to a difficult situation and face painful experiences, chronic depression is harmful.

The hierarchical linear model (HLM) is the preferred method to analyze data on depression[20,21,22]. The HLM statistical method of research analysis is chosen for this study for the following reasons: (1) When data analysis occurs within a nested structure, and higher-level variables affect lower-level variables in a hierarchical structure, the results of traditional regression analysis or analysis of variance will be skewed; and (2) in traditional regression analysis or analysis of variance, the data are collected at the group level, and each group must contain the same characters before analysis in order to avoid the appearance of different character clusters. Cluster analysis before results analysis prevents skewed results, but does not provide further explanations of the data. The HLM can simultaneously process factors at different levels of the analysis, and progressively break them down by variance, making possible cause and effect inferences. Its submodels have nested relationships, and the HLM has the ability to compare submodels.

The HLM can also establish different level models according to the characteristics of the hierarchical structure of the research. It displays the structure of each level and computes effects individually and by the variation in each level. In other words, the HLM allows researchers to carry out hypothesis testing among and between different levels of analysis. To summarize, when the data have a hierarchical structure, or when it is assumed that the various levels of the analysis are interacting, the HLM should be used to carry out the data analysis.

## METHODS

### Participants

The participants in this study were 411 classes totaling 3,582 students (1,694 girls and 1,888 boys) and 172 teachers, during the fall, 2004 semester from ten different public junior high schools in Taipei, Taiwan. Class size varied from 30–40 students aged 13–15 years. Because the study collected data from the student level and the teacher level, the HLM was adopted for the cross-level analysis. To match 172 valid teacher-level questionnaires, 1,479 valid corresponding student-level questionnaires were chosen. The investigation was conducted in November and December of 2004. To ensure that students could freely express their opinions, students responded anonymously to the questionnaire and teachers were absent during the evaluation period.

The Zhongxiao Branch of the Taipei City Hospital enforces a community public health team project from the Department of Health in Taiwan Government. One of the objectives of this project is to realize the situation about community adolescent depression. Therefore, the hospital had telephone interviews with student counseling directors responsible for the junior high schools located in the Districts of Nangang, Neihu, Xinyi, Sungshan, and Daan in the city of Taipei. The directors were asked about their willingness to participate in the questionnaire study and ten schools agreed to participate. Random sampling resulted in the selection of ten students from each class from ten schools. To control the differences between public and private schools, only public schools were studied. Selecting only public schools created a representative sample because public schools constitute the majority of schools in Taipei.

Of the 4,110 questionnaires distributed, 3,723 were returned, 141 of which were invalidated, amounting to a return of 3,582 questionnaires, or a valid questionnaire return rate of 87.2%. Each teacher in charge of a class was given a questionnaire. Of the 411 teacher questionnaires distributed, 235 were returned, of which 63 were invalidated, amounting to a total of 172 questionnaires, or a valid questionnaire return rate of 41.8%. The reasons for the lower teacher questionnaire return rate could be that some teachers were unwilling to participate or some teachers did not state the class they were in charge of, making it impossible to match the teacher questionnaire with the class questionnaire.

Because this study seeks to explore how teacher behavior influences student depression, data were collected from the student level and the teacher level, and the HLM was adapted for the cross-level analysis. To match 172 valid teacher-level questionnaires, 1,479 valid corresponding student-level questionnaires were chosen. It is understood that there are many factors influencing student depression in Taiwan, including the nature of the distinct educational system, teacher procedures in classroom management, and the influence on student exceed other non-Chinese culture teacher. It is because the requirement of standardized high school and college exam, a lot of pressure is on the students to perform well in order to get into top schools. Moreover, there is more hierarchical relationship between teachers and students in Chinese culture than other cultures, hence this study aimed at particular Taiwan education conditions exploring the teacher's behavior as it influences student depression. This study investigates the Taiwanese educational situation in particular to explore the influence of the teacher on student expression.

## The Questionnaire

Teacher depression was assessed using the Beck Depression Inventory-II (BDI)[23,24]. The BDI consists of 21 question items having 4 alternatives ranging from 0 (low depression) to 3 (maximum depression), with total scores ranging from 0–63. The survey prompted the user to answer such questions, as "I am disappointed in myself". The BDI is the most prominent and frequently cited self-report measure of depression[25]. It has been used in numerous studies with varying norms for different groups[26,27]. Student depression was assessed using the Children's Depression Inventory (CDI) consisting of 27 items (e.g., "I am sad all the time")[28]. The CDI was designed to parallel the BDI, but was adapted to suit the developmental- and reading-level needs of children. It contains only three statements per item; hence, scores on each of its 27 items can range from 0–2, with total scores ranging from 0–54[25]. The Zhongxiao Branch of the Taipei City Hospital bought the BDI copyright from the Chinese Behavioral Science Corporation of Taiwan. The CDI[29] was obtained with authorization from Dr. M. Kovacs and Multi-Health Systems, Inc. Both surveys were double-translated to Chinese, and the final Chinese scale had a Cronbach's $\alpha$ of 0.86 and retest reliability of 0.85.

Student perceptions of teacher emotional overinvolvement referenced concepts developed by Coyne et al.[4] and Docherty and Serper[12] in drawing up the survey items. It consists of 11 items (e.g., "Teacher has extremely strong expectations for me"). All items in the pretest were found to have good measurement properties ($\alpha$ = 0.82), so were adopted into in the formal study. Student core self-evaluations were assessed using the core self-evaluations scale developed by Timothy et al.[30]. The self-evaluation used four traits well established in the literature: (1) self-esteem, (2) generalized self-efficacy,

(3) neuroticism, and (4) locus of control. It consisted of 12 items with α = 0.82. An example of the items used was "Overall, I am satisfied with myself." The scale of teacher involvement was assessed using the personal involvement inventory from Zaichkowsky[31]. The ten items achieved Cronbach's α of 0.89, meaning they adequately represented the concept of involvement. Sample items include the following: "Do you feel classroom management is important?"(α = 0.89).

Previous studies showed that one of the factors affecting student depression is family support[32] and, therefore, in this study, its influence on student depression was measured. Measurement of family support consisted of two items (e.g., "When you are in a bad mood, can you find family to stay with you?"). Personal performance satisfaction, also shown by previous studies to affect student depression, was tested by one item (e.g., "Are you satisfied with your performance this semester?"). Student perception of teacher support was assessed using the supervisor support scale developed by Alex et al. in 2003[33]. The scale consisted of three items with α = 0.80. An example of the items included was "I find my teacher very helpful in my school life." Teacher support was defined as student belief that the teacher offered them study-related assistance to aid in their educational performance. The evaluation form also provided information on the background of students and teachers. The background section identified the educational background of parents, student grades, and days absent[34,35,36,37,38].

## Research Hypotheses

Based on the purpose of this study and the literature review, the following hypotheses were formulated:

Hypothesis 1: The level of depression will vary significantly between students in different classes.

Hypothesis 2: Teacher involvement, teacher depression, and personal background (age, educational background, marital status, and number of children) have a significant influence on student depression.

Hypothesis 3: Student perceptions of teacher emotional overinvolvement and support in general (teacher and family support), core self-evaluations, and student personal background (personal performance satisfaction, parental educational background, school grades, and days absent) have a significant influence on student depression.

Hypothesis 4: The level of teacher involvement, teacher depression, and personal background (age, educational background, marital status, and number of children) will moderate the relationship between student-perceived teacher emotional overinvolvement, core self-evaluations, student-perceived support in general (teacher support and family support), and student personal background (personal performance satisfaction, parental educational background, school grades, and days absent) and student depression.

## RESULTS

This study used SPSS 11.0 and HLM 5.05 statistical software to perform the data analysis. All statistical tests used 0.05 significance for gauging a type I error.

Of a total of 172 teachers, 39 were men and 133 were women. For teacher involvement, the average involvement value was 4.02 with a standard error of 0.73. For teacher depression, one teacher scored above 31, 16 teachers between 21 and 30, 10 teachers between 17 and 20, and 21 teachers between 11 and 16, and 124 teachers scored under 11, for a total of 48 teachers affected by depression. Of a total of 1,479 students, 699 were female and 780 were male. For student depression, 4 students scored 41 or above, 39 students scored between 31 and 40, 264 students scored between 21 and 30, 301 students scored between

16 and 20, and 871 students scored under 20, for a total of 608 students affected by depression. For student perception of teacher emotional overinvolvement, the average involvement value was 2.88, with a standard error of 0.91. For core self-evaluations, the average value was 3.74 with a standard error of 0.83.

## Construct Validity

The following analyses were performed to demonstrate the construct validity of the student depression survey tool. The criterion-related validity of the student depression measure was analyzed by examining its relationship to other measures to which it should be theoretically related. As reported in Table 1, the pattern of correlations was consistent with the aforementioned measures. At the student-level, student depression was significantly correlated with student perception of teacher emotional overinvolvement (r = 0.262, $p < 0.01$), teacher support (r = 0.290, $p < 0.01$), core self-evaluations (r = –0.645, $p < 0.01$) , family support (r = –0.472, $p <$ 01), parent educational background (r = –0.106, $p < 0.01$), school grades (r = –0.254, $p < 0.01$), days absent (r = –0.092, $p < 0.01$) , and personal performance satisfaction (r = –0.354, $p < 0.01$). As these relationships were largely consistent with theories about, and empirical evidence for, student depression, the results provided criterion-related validity for evidence of the student depression.

**TABLE 1**
**Descriptive Statistics and Correlations**

| Variable | Means | SD | 1 | 2 | 3 | 4 | 5 | 6 | 7 | 8 | 9 |
|---|---|---|---|---|---|---|---|---|---|---|---|
| Student-Level | | | | | | | | | | | |
| Student depression | 14.30 | 7.99 | | | | | | | | | |
| Emotional overinvolvement | 2.88 | 0.91 | 0.262** | | | | | | | | |
| Teacher support | 3.36 | 1.21 | 0.290** | 0.267** | | | | | | | |
| Core self-evaluations | 3.74 | 0.83 | –0.645** | –0.240** | –0.243** | | | | | | |
| Family support | 3.50 | 1.28 | –0.472** | –0.162** | –0.246** | 0.428** | | | | | |
| Parent educational background | 3.20 | 0.83 | –0.106** | –0.018 | 0.006 | 0.124** | 0.209** | | | | |
| School grades | 3.34 | 1.43 | –0.254** | –0.151** | –0.051* | 0.235** | 0.259** | 0.357** | | | |
| Days absent | 3.72 | 0.88 | –0.092** | –0.050 | –0.052* | 0.161** | 0.243** | 0.262** | 0.274** | | |
| Personal performance satisfaction | 3.36 | 1.42 | –0.354** | –0.091** | –0.166** | 0.438** | 0.366** | 0.149** | 0.201** | 0.217** | |
| Teacher-Level | | | | | | | | | | | |
| Teacher involvement | 4.02 | 0.73 | | | | | | | | | |
| Teacher depression | 7.93 | 7.98 | –0.226** | | | | | | | | |
| Age | 2.03 | 0.96 | 0.007 | –0.122 | | | | | | | |
| Educational background | 1.74 | 0.88 | 0.096 | –0.08 | 0.331** | | | | | | |
| Marital status | 3.34 | 1.14 | 0.074 | –0.014 | 0.597** | 0.365** | | | | | |
| Number of children | 1.01 | 0.99 | –0.004 | –0.04 | 0.562** | 0.092 | 0.407** | | | | |

*Note:* * $p < 0.05$; ** $p < 0.01$; two-tailed tests.

## Aggregation of Variables

Hierarchical linear modeling[20,21,22] was used for the cross-level analysis. Because the key dependent variable of this study, student depression, was measured via student self-reporting, the construct validity of this measure was assessed by examining its dimensionality (Cronbach's α), and criterion-related validity. The viability was then confirmed by examining the within-group agreement ($\gamma wg$)[39,40], intraclass correlation (ICC(1))[41,42], and reliability of the mean (ICC(2))[41,42]. $\gamma wg$ values were computed for these variables and a median value of 0.947 was obtained for teacher involvement, 0.726 for student-perceived teacher emotional overinvolvement, 0.817 for core self-evaluations, and 0.547 for teacher support. In addition to teacher support, other $\gamma wg$ values were well above the conventionally acceptable value of 0.70. The following ICC(1) and ICC(2) values were obtained: teacher involvement, 0.043 and 0.435; student-perceived teacher emotional overinvolvement, 0.210 and 0.696; core self-evaluations, 0.155 and 0.612; and teacher support, 0.228 and 0.718. All of these values were comparable to the median or recommended ICC values reported in the literature[41,42]. It was thus concluded that aggregation was justified for these variables.

## Hierarchical Linear Model Results for the Antecedents of Student Depression

### Null Model

The hypotheses of this study predicted that both student-level and teacher-level variables would be significantly related to student depression. In order for these hypotheses to be supported, there had to be a significant relationship in the variance in student depression between classes. Thus, using the HLM, a null model was estimated in which no predictors were specified for either the level-1 or level-2 function to test the significance level of the level 2 residual variance of the intercept ($\tau 00 = 2.643$, $p < 0.01$) reported in Table 2A. The ICC(1) was 0.041, indicating 4.1% of the variance in student depression resided between classes, and 95.9% of the variance resided within classes. Therefore, Hypothesis 1 was supported.

**TABLE 2A**
**One-Way ANOVA with Random Effects**

| Fixed Effects | | Coefficient | Standard Error | T-ratio | *p*-value |
|---|---|---|---|---|---|
| Grand mean of student depression $\gamma$ 00 | | 14.305 | 0.239 | 59.811 | 0.000 |
| **Random Effects** | **SD** | **Variance Component** | **df.** | **Chi-square** | ***p*-value** |
| Random effects associated with teacher-level $U_0$ | 1.626 | 2.643 | 171 | 233.811 | 0.001 |
| Random effects associated with student-level R | 7.820 | 61.156 | | | |

### Teacher-Level Predictors

To test Hypothesis 2, the intercept coefficients obtained from level 1 were regressed on the measures of teacher-level involvement, teacher depression, and teacher personal information at level 2. As reported in Table 2B, teacher educational background ($\gamma 02 = 0.704$, $p < 0.05$) demonstrated a significant relationship with student depression. As a group, the specified teacher-level variables accounted for 7.9% of the between-class variance in student depression.

**TABLE 2B**
**Means as Outcome Regression**

| Fixed Effects | | Coefficient | Standard Error | T-ratio | *p*-value |
|---|---|---|---|---|---|
| Grand mean of student depression γ00 | | 14.324 | 0.228 | 62.557 | 0.000 |
| Age, γ01 | | –0.543 | 0.318 | –1.703 | 0.088 |
| Educational background, γ02 | | 0.704 | 0.313 | 2.249 | 0.024 |
| Marital status, γ03 | | 0.341 | 0.321 | 1.063 | 0.288 |
| Number of children, γ04 | | –0.185 | 0.251 | –0.737 | 0.461 |
| Teacher involvement, γ05 | | –0.134 | 0.309 | –0.436 | 0.663 |
| Teacher depression, γ06 | | –0.044 | 0.030 | –1.434 | 0.152 |
| **Random Effects** | **SD** | **Variance Component** | **df.** | **Chi-square** | ***p*-value** |
| Random effects associated with teacher-level *U*0 | 1.559 | 2.432 | 164.000 | 218.500 | 0.003 |
| Random effects associated with student-level R | 7.818 | 61.121 | | | |

## *Student-Level Predictors*

The hypotheses predicted that student-level variables will be associated with student depression. A level-1 model including these variables with no predictors specified for the level-2 model was estimated. As reported in Table 3A, the variables explained 57.9% of the within-class variance. Specifically, student-perceived teacher emotional overinvolvement ($\gamma 10 = 0.827$, $p < 0.001$), teacher support ($\gamma 20 = 0.411$, $p < 0.05$), core self-evaluations ($\gamma 30 = -4.432$, $p < 0.001$), family support ($\gamma 40 = -1.270$, $p < 0.001$), school grades ($\gamma 60 = -.605$, $p < 0.001$), and days absent ($\gamma 70 = .561$, $p < 0.05$) showed a significant relationship to student depression. Therefore, some aspects of Hypothesis 3 were supported. However, personal performance satisfaction and parental educational background were not significantly related to student depression.

**TABLE 3A**
**Random Coefficients Regression Model**

| Fixed Effects | Coefficient | Standard Error | T-ratio | *p*-value |
|---|---|---|---|---|
| Level-1 Intercept, γ00 | 14.320 | 0.239 | 59.784 | 0.000 |
| Emotional overinvolvement, γ10 | 0.827 | 0.154 | 5.343 | 0.000 |
| Teacher support, γ20 | 0.411 | 0.186 | 2.208 | 0.027 |
| Core self-evaluations, γ30 | –4.432 | 0.235 | –18.855 | 0.000 |
| Family support, γ40 | –1.270 | 0.138 | –9.154 | 0.000 |
| Parent educational background, γ50 | 0.004 | 0.218 | 0.022 | 0.983 |
| School grades, γ60 | –0.605 | 0.127 | –4.759 | 0.000 |
| Days absent, γ70 | 0.561 | 0.271 | 2.064 | 0.039 |
| Personal performance satisfaction, γ80 | –0.207 | 0.135 | –1.529 | 0.126 |

*Note:* Expect for $U_{0j}$, *p*-value of random effects associated with class $U_{ij}$ almost more than 0.05.

## Testing Cross-Level Interactions

A prerequisite for testing cross-level interactions was that there is significant random variance for the variables in the intercepts-as-outcomes models estimated in the previous step. As reported in Table 3B, Fig.1, and Fig. 2, in which estimates of the random-variance components appear in parentheses, teacher involvement ($\gamma 16 = 0.430$, $p < 0.05$) and teacher depression ($\gamma 17 = 0.048$, $p < 0.05$) were significantly variable at the level-1 student-perceived teacher emotional overinvolvement relationship to student depression across classes; teacher involvement ($\gamma 36 = 0.790$, $p < 0.05$) was significantly variable at the level-1 core self-evaluations relationship to student depression relationship across classes. Therefore, some aspects of Hypothesis 4 were supported.

**TABLE 3B**
**Intercept- and Slopes-as-Outcomes Model**

| Fixed Effects | Coefficient | Standard Error | T-ratio | P-value |
|---|---|---|---|---|
| **Grand Mean of Student Depression** | | | | |
| Base, $\gamma 00$ | 14.325971 | 0.230682 | 62.103 | 0.000 |
| Educational background, $\gamma 02$ | 0.695849 | 0.314442 | 2.213 | 0.027 |
| **Emotional Overinvolvement** | | | | |
| Base, $\gamma 10$ | 0.807257 | 0.165593 | 4.875 | 0.000 |
| Teacher involvement, $\gamma 16$ | 0.430634 | 0.213219 | 2.020 | 0.043 |
| Teacher depression, $\gamma 17$ | 0.048454 | 0.023820 | 2.034 | 0.042 |
| **Core Self-Evaluations** | | | | |
| Base, $\gamma 30$ | –4.520996 | 0.224124 | –20.172 | 0.000 |
| Teacher involvement, $\gamma 36$ | 0.790047 | 0.344859 | 2.291 | 0.022 |

*Note:* The table lists only variables with a *p*-value of less than 0.05. *p*-value of random effects associated with class $\underline{U}_{ij}$ are all more than 0.05.

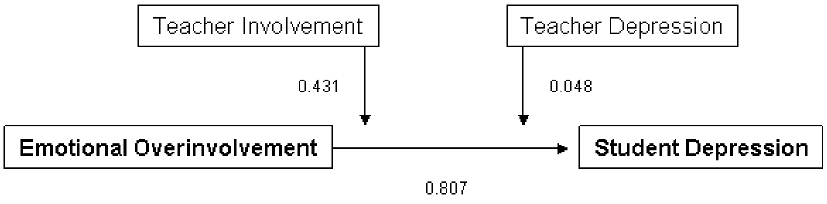

**FIGURE 1**. Emotional overinvolvement to student depression: cross-level interactions.

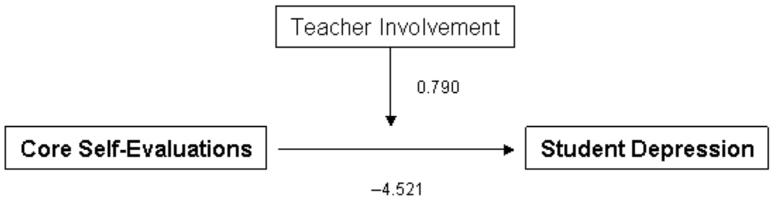

**FIGURE 2**. Core self-evaluations to student depression: cross-level interactions.

## DISCUSSION

Based on previous results with the null model, different classes of students show significant differences in student depression. According to a new Taiwanese statute established in 1997, school shall not divide students into different class groupings based on their academic performance. Variation in the academic ability of the students may not account for the differences in depression between different classes. This indicates that the individual characteristics of each class, such as classroom management style or teacher instructional philosophies, will significantly impact student depression. Such differences may explain the support for Hypothesis 1 in the present study. The results of this study indicate that 4.1% of the variance in student depression resides between classes, and 95.9% of the variance resides within classes. Although the teacher-level variables are found to have a significant influence on student depression, student-level variables are far more important than teacher-level variables in explaining student depression.

The results indicate that 7.9% of the variance in student depression resided in teacher involvement, teacher depression, and teacher personal background between classes, and 92.1% of the variance resided within variables this model cannot explain. Furthermore, the variance of random effects in level 2 shows that after eliminating the effects of the explanatory variables in level 2, student depression in various classes is significantly different, implying that other variables are necessary to explain student depression. Research on these other variables is recommended as a topic for further study. Teacher educational background is positively and significantly correlated with student depression ($\gamma 02 = 0.704$, $p < 0.05$). In other words, the more educated the teacher, the more depressed the students. This probably results from higher teacher expectations for student performance, and correspondingly greater pressure placed on students.

Additionally, the analysis has indicated that the higher the student perception of teacher emotional overinvolvement, the higher the level of student depression. When a teacher overmanages the classroom, students perceive pressure, resulting in high scores on depression indices. Interestingly, the analysis also has shown that the higher level of teacher support, the higher level of depression. Students indicated that teacher support is experienced as pressure to live up to expectations. Absences are also positively related to student depression, as they disrupt peer and teacher relationships and lead to large amounts of work that must be done at home. Students with lower student core self-evaluations perceive higher pressure, leading to increased levels of student depression. Lower student family support is also positively correlated with higher levels of student depression. Because grades are tightly linked to advancement to good schools in Taiwan, the lower the student grades, the higher the level of student depression. Overall, our results indicate that 57.9% of the variance in student depression resided at the student-level.

Finally, teacher involvement, teacher depression, and teacher personal background are found to affect student depression. The $\gamma 16 > 0$ in student-perceived teacher emotional overinvolvement influence on student depression appears to indicate that the higher the level of teacher involvement in classroom management, the higher the student perception of teacher emotional overinvolvement, which triggers higher levels of student depression. Higher levels of teacher depression have the same effect when the teacher is perceived to be emotionally overinvolved. The case is similar for the effects of student perceptions of teacher emotional overinvolvement. The result of $\gamma 36 > 0$ indicates that core self-evaluations affects student depression when there is higher teacher involvement in classroom management. When students perceive overinvolvement as a form of pressure, student core self-evaluations decrease, triggering higher levels of student depression.

## CONCLUSION

The results of this study suggest that the classroom atmosphere needs to be constructed carefully to ensure that students adapt well to school life. Because the emotional state of the teacher is an important factor in the development of student depression, teachers should be monitored to ensure that they do not become depressed, and therapy should be provided to teachers suffering from depression.

Positive student subjective consciousness can help students better adapt to school circumstances. The following methods are recommended to enhance student well-being: (1) Homeroom teacher should make use of various strategies that unite and order the class to increase student satisfaction and decrease class conflict. Teacher behavior should only indirectly affect behavior and the teacher should employ counseling strategies to enhance student self-esteem. (2) Instruction should be provided to students and teachers to help them adopt more positive subjective opinions of themselves and to help them adapt to changing circumstances. Teachers should be instructed in how their behavior can affect student well-being and performance. Since student subjective consciousness is a particularly important factor affecting student depression, it is very important to aid students and teachers in increasing positive student consciousness.

According to a Taipei study, half of the high school teachers are susceptible to depression and more than sixty five percent feel pressured[43]. Data such as these, along with the present study, suggest that the government should establish a comprehensive teacher mental health management system. This includes counseling for stress and relationships through marriage, group, and individual therapies, as well as through offering mental health courses. Similarly, students should also be taught stress management techniques and provided with information on achieving well-being.

Our study was limited in time and space. More research is needed on variation among different communities in Taiwan, and over long periods of time. The students were minors with undeveloped self-awareness. A survey cannot adequately capture a rich description of their experiences of impression. We propose that further research use quantitative and qualitative analytical methodologies to triangulate the data and develop a more robust description of the relationship between student depression and teacher overinvolvement. Further, our study shows that many other variables must account for student depression, and investigation is needed into what such variables might be and how they might interact.

## BIOSKETCHES

**Fen Fen Huang, MPH,** Department of Business Administration, National Taiwan University of Science and Technology, Taiwan. E-mail: wa-0147@mail.taipei.gov.tw

**Cou Chen Wu, PhD,** Department of Business Administration, National Taiwan University of Science and Technology, Taiwan. E-mail: wu@ba.ntust.edu.tw

**Chang Ya Hu, PhD,** Department of Business Administration, National Taiwan University of Science and Technology, Taiwan. E-mail: cyhu@ba.ntust.edu.tw

**Sun Shen Yang, MD, MPH,** Department of Community Medicine, Center for Occupational Disease, Taipei City Hospital, Taiwan. E-mail: DAH48@tpech.gov.tw
